# Rapid synthesis of vertically aligned α-MoO_3_ nanostructures on substrates†

**DOI:** 10.1039/d0ra01281e

**Published:** 2020-06-24

**Authors:** Sohaila Z. Noby, Ka Kan Wong, Ananthakumar Ramadoss, Stephan Siroky, Matthias Hagner, Klaus Boldt, Lukas Schmidt-Mende

**Affiliations:** Department of Physics, University of Konstanz 78457 Konstanz Germany lukas.schmidt-mende@uni-konstanz.de; National Research Centre NRC, Department of Solid State of Physics 12622 Cairo Egypt; SARP-LARPM, Central Institute of Plastic Engineering and Technology (CIPET) 751024 Bhubaneswar India; Department of Chemistry, University of Konstanz 78457 Konstanz Germany; Department of Chemistry & Zukunftskolleg, University of Konstanz 78457 Konstanz Germany

## Abstract

We report a new procedure for large scale, reproducible and fast synthesis of polycrystalline, dense, vertically aligned α-MoO_3_ nanostructures on conducting (FTO) and non-conducting substrates (Si/SiO_2_) by using a simple, low-cost hydrothermal technique. The synthesis method consists of two steps, firstly formation of a thermally evaporated Cr/MoO_3_ seed layer, and secondly growth of the nanostructures in a highly acidic precursor solution. In this report, we document a growth process of vertically aligned α-MoO_3_ nanostructures with varying growth parameters, such as pH and precursor concentration influencing the resulting structure. Vertically aligned MoO_3_ nanostructures are valuable for different applications such as electrode material for organic and dye-sensitized solar cells, as a photocatalyst, and in Li-ion batteries, display devices and memory devices due to their high surface area.

## Abstract 2

We report a procedure for large scale, reproducible and fast synthesis of polycrystalline, dense, vertically aligned α-MoO_3_ nanostructures on conducting (FTO) and non-conducting substrates (Si/SiO_2_) by using a simple, low-cost hydrothermal technique.

## Graphical abstract

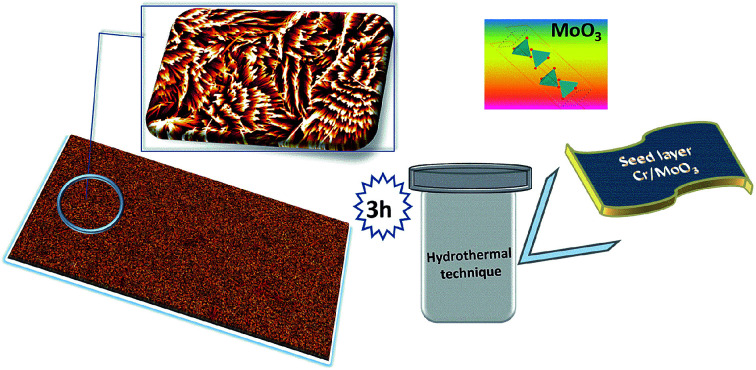

## 1 Introduction

Over the last decade, transition metal oxides with reduced dimensionality (2D, 1D, 0D) have gained attention as a building block for various hierarchical micro and nanostructures in smart, functional materials due to their excellent physical, chemical, electrical and optical properties.^1^ Molybdenum trioxide (MoO_3_) as a wide band gap material occupies a prominent position among other transition metal oxides, owing to its distinctive properties such as 2D layered structure and not completely filled d-orbitals. MoO_3_ can be employed in a wide range of applications such as organic solar cells,^2^ Li-ion batteries where 2D layered MoO_3_ material is currently seen as the strongest competitor against graphene,^3,4^ heterogeneous catalysis,^5^ chemical^6^ and bio-sensors,^7^ water splitting^8^ and memory devices.^9^ MoO_3_ exists in three different crystal structures, orthorhombic (α-MoO_3_), monoclinic (β-MoO_3_), and hexagonal (h-MoO_3_). The α-MoO_3_ crystal structure is a thermodynamically stable layered structure with *Pbnm* symmetry. It consists of distorted [MoO_6_]^6+^ octahedra, which are connected in a zig–zag chain *via* edges along the [100] direction and linked *via* corners in *trans* position in the [001] direction, forming double layers along the [010] direction.^10^ h-MoO_3_ is formed from the same [MoO_6_]^6+^ zig–zag chains, which are linked by corners in *cis* position forming a tunnel structure.^11^

The synthesis of various MoO_3_ nanostructures has been achieved through different methods,^12^ including electro-deposition,^13^ thermal evaporation,^14^ chemical vapour deposition,^15^ sonochemical methods,^16^ flame,^17^ aqueous solution processes,^18^ laser-assisted evaporation,^19^ and ultrasonic spray pyrolysis.^20^ The morphological engineering development is owing to the unique characteristics of large surface area, the small dimensions comparable to the Debye length, superior stability, and ease functionalization, which differ from the respective bulk properties.^21,22^ However, growing dense, vertically aligned 1D MoO_3_ nanostructures in a reproducible manner remains a big challenge. Vertically aligned 2D and 1D nanostructures films were found to enhance the sensitivity of gas sensing materials in comparison to a random network of 2D materials on a substrate.^23–25^ Recently, highly efficient, low power consuming, and long-lasting vertically aligned nanowire-based multifunctional devices have been demonstrated in different applications such as MEMS-based sensors and flexible wearable and portable safety clothes^26^ and multisensor chips (optical, magnetic and conductometric gas sensor).^27^ Vertically aligned metal oxides have been integrated into organic solar cells, which showed improved charge separation and extraction.^28^ The aforementioned synthesis procedures for vertically aligned 1D and 2D MoO_3_ rely on a range of sophisticated techniques such as using thermal evaporation at very high boat temperature of 1100 °C to prepare the structures on Si substrates,^29^ plasma-assisted paste sublimation on nickel coated glass,^30^ and also modified hotplate method at 450 °C on Au/Si substrates.^31^ Those methods require high temperature or are limited to certain types of substrates. Furthermore, the final product is neither highly reproducible nor does it provide a very dense structure, but only a low number of wires or flakes per unit area. The hydrothermal process is the most versatile and cheapest technique employed in nanochemistry to grow a variety of nanostructures. It has been used intensively to synthesise MoO_3_ nanostructures as a powder or dispersion. Additionally, the hydrothermal technique has been used to grow direct 2D and 1D structures of other metal oxides on substrates such as ZnO, TiO_2_ and SnO_2_. However, we are not aware of such synthesis directly on substrates for MoO_3_.

An essential goal of this work is to grow vertically aligned, densely packed MoO_3_ structures as films on substrates such as transparent conducting oxides (TCOs) or Si. Here, we report specifically an efficient method to produce a homogeneous and dense, vertically aligned film of two-dimensional α-MoO_3_ nano-blades on seeded substrates using a highly reproducible, hydrothermal method with, short reaction. The synthesis proceeds without the use of any additional organic surfactants, catalysts, or dispersing additives. Here, we studied the various parameters such as reaction time, precursor concentration, and pH value to control the growth, the morphology and phase evolution of the MoO_3_ nanostructure.

## 2 Experimental section

### 2.1. Chemicals

All materials included in our study have used as received and used without further purification. For the precursor solution, sodium molybdate dihydrate (99.5%, Sigma Aldrich), and hydrochloric acid (37%, VWR chemicals) were used. As evaporation sources for the seed layer molybdenum(vi) oxide powder (99.98%, Sigma Aldrich) and chrome plated tungsten rods (99.9%, Testbourne) were used.

### 2.2. MoO_3_ seed layer formation/coating

Different types of substrates such as commercial fluorine-doped tin oxides (FTO) (15 Ω cm^−2^), p-doped Si/SiO_2_ wafers (SiO_2_ thickness: 1 μm) were cleaned using a standard procedure by sonication in acetone (5 min), isopropanol (5 min), and ethanol (15 min), followed by drying in a nitrogen flow. The cleaned substrates were treated with a UV/ozone cleaner (Ossila) for 20 min to form a polar surface to improve the adhesion of the MoO_3_ seed layer.^32^ The MoO_3_ seed layer was deposited on the different substrates using thermal evaporation (Tectra mini-coater) at a base pressure of <2 × 10^−6^ mbar with applied current and power of 15 A and 1 kW, respectively. The thickness of the film was monitored using a quartz micro-balance with a resolution of ±0.1 nm. Finally, the seed layer coated substrate was annealed at 450 °C for 1 h under ambient atmosphere.

### 2.3. Vertically aligned of MoO_3_ nanostructures growth process

An aliquot of concentrated hydrochloric acid (HCl) was added dropwise to an aqueous solution of Na_2_MoO_4_·2H_2_O into a PTFE autoclave liner to yield a total volume of 21.5 mL. The mixed solution was stirred for at least 10 min to dissolve the precursors completely. Then, the seed layer coated substrates were placed upright into a PTFE holder and submerged in the prepared precursor solution. Finally, the PTFE autoclave liner was sealed and put into a pre-heated oven at 180 °C for 210 min. After the reaction, the autoclave was rapidly cooled down to ambient temperature in a water bath. The grown MoO_3_ substrates were washed with ethanol and allowed to dry under ambient conditions.

### 2.4. Characterization

X-ray diffraction (XRD) analysis was performed with a Bruker AXS D8 Advance diffractometer employing a Bragg–Brentano geometry and Cu-K_α_ radiation and using a grazing incidence geometry (GIXRD). Surface imaging was done using Zeiss Cross Beam 1540XB field emission scanning electron microscope (FE-SEM) using an acceleration voltage of 5 kV. High-resolution transmission electron microscope (HR-TEM) images were obtained on a JEOL JEM-2200FS microscope operated at 200 kV. The HR-TEM samples were prepared on carbon-coated copper grids supplied by Quantifoil GmbH. The obtained nanostructures were analyzed with selected area electron diffraction (SAED), bright-field imaging, and zero-loss filtered high-resolution TEM.

## 3 Results and discussion

**Fig. 1:**
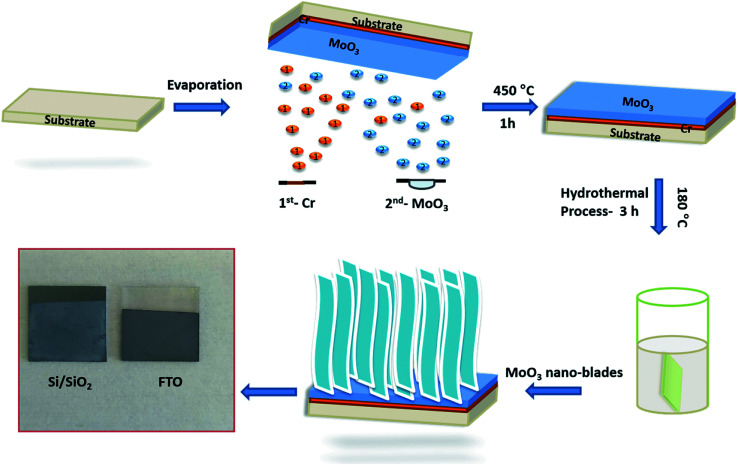
Schematic illustration of synthesis process of vertically aligned α-MoO_3_ nano-blades.

A dense layer of vertically aligned MoO_3_ nano-blades was prepared on various substrates through a process, which consists of first depositing the seed layer, followed by hydrothermal synthesis of the MoO_3_ film. The whole synthesis process is schematically represented in Fig. 1.

### 3.1. Influence of adhesive and seed layers on the growth of vertically aligned MoO_3_ nano-blades

To ensure proper adhesion of the nanostructures on the substrate, a thin adhesion layer has to be deposited initially. We found that only Cr worked well as adhesion layer for the MoO_3_ growth.

**Fig. 2:**
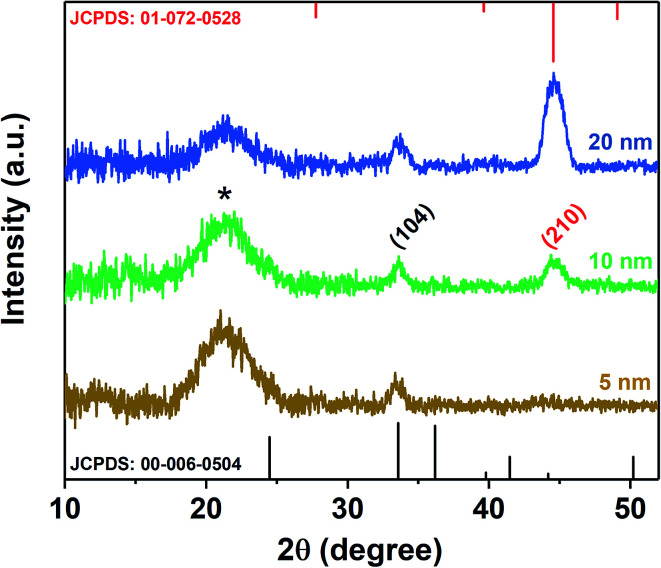
GIXRD patterns of annealed chromium layer on Si/SiO_2_ substrates, with corresponding stick patterns of Cr_2_O_3_ (black) and Cr_3_O (red), respectively. The star indicates the amorphous SiO_2_ peak.

The structure and composition of the Cr layer were characterized using GIXRD at a grazing incidence angle of 0.5°. In Fig. 2, the XRD patterns of the Cr adhesive layer with thicknesses of 5, 10 and 20 nm are presented. The Cr-layer was directly exposed to an annealing process (450 °C for 1 h) to ensure the same condition, which has been applied to the complete seed layer, including the evaporated MoO_3_ layer on top of the Cr layer. The obtained XRD pattern revealed that the coated metallic Cr was converted to chromium oxides consisting of the two most stable chromium oxides phases: rhombohedral Cr_2_O_3_ and cubic Cr_3_O. Oxidation of the Cr layer can be attributed to interaction with the oxygen-rich substrate on the bottom, as well as the formation of the metal oxide layer from the top during the annealing process.^33^ The stoichiometry changed in favour of Cr_3_O in thicker adhesive layers, indicated by the increasing intensity of the (210) reflex at 44.55° as a predominant peak of Cr_3_O (space group *Pm*3̄*n*, JCPDS 01-072-0528). An earlier study has shown that this phase of Cr oxide is formed from the interaction between Cr_2_O_3_ and metallic Cr.^34^ In the case of thinner layers (5 nm) Cr_3_O cannot be detected. Cr_2_O_3_ was indicated by the (104) reflex at 33.58° (space group *R*3̄*c*, JCPDS 00-006-0504) and this result is consistent with previous studies, which indicate conversion of Cr to its oxides by applying heat treatment and/or exposure to an oxygen-rich atmosphere.^35,36^ The broad peak observed in all films centred at 21.4° (indicated by “*”) is attributed to the amorphous SiO_2_ substrate (JCPDS 01-086-1561).^37^ It was demonstrated that the chromium layer forms very strong chemical bonds to the oxidized substrate (SiO_2_, FTO) *via* an oxide transition layer.^38,39^ In the seminal study by Benjamin *et al.* reveals that the adhesion of the Cr layer on glass substrates is enhancing with the time. In particular, it affirms a better adhesion compared to nobel metals, strongly depending on the deposition condition and exposure to air.^38,40^ The thickness of the Cr oxide layer was found to be influenced by the temperature during oxygen exposure and saturates at 400 °C.^36^ Furthermore, prior studies^33^ have shown that using Cr as an adhesion layer for Au/dielectric and semiconductor materials on glass and oxidized substrates decreased the roughness of the Au layer, which leads to smaller and more stable nucleation centres. Best adhesion was reported for thicknesses between 1 and 30 nm. Thicker films up to 100 nm lead to stress, which causes delamination.^41^ Benjamin *et al.* showed that the Cr layer was composed of closely spaced islands with a width of 5 nm, and a height of 10 nm,^38^ which is consistent to our observation (see ESI, Fig. S1†). We suggest that beyond acting as an adhesion layer, the Cr oxide could also function as nucleation centres. The atomic radii of Cr and Mo are similar, which allows the adhesion layer to reduce the lattice mismatch between α-MoO_3_ and the substrate. The effect can be quantified by comparing the lattice misfit (*f*) between the exposed lattice planes according to XRD calculated through the relation: MoO_3_/FTO: 16%, MoO_3_/SiO_2_: 21%, Cr_2_O_3_/FTO only 6%, Cr_2_O_3_/SiO_2_ as little as 1%, Cr_3_O/FTO: 4% and Cr_3_O/SiO_2_: 9%, which are calculated through theoretical bulk values (see ESI, Table S1,† for used lattice parameters):^42^1
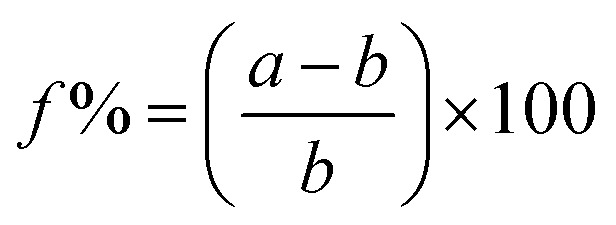
where *a* and *b* are the in-plane lattice parameters (preferred orientation) of the deposited layer and substrate, respectively. These observations are in line with previous studies and form a compelling argument for the use of a Cr adhesion layer for fast growth of the MoO_3_ nano-blades.

**Fig. 3:**
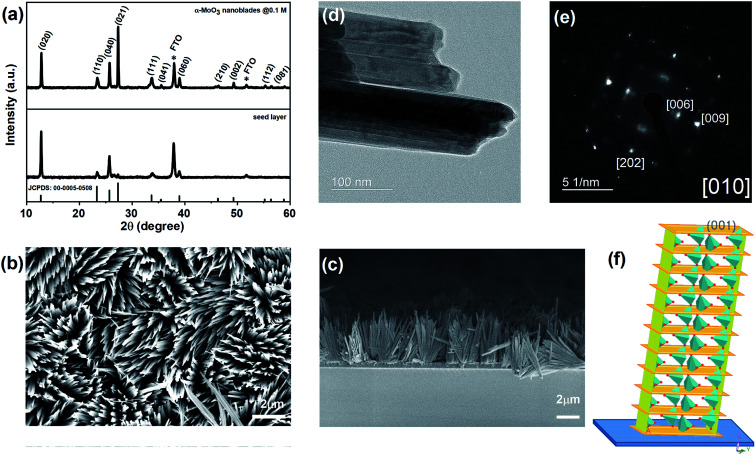
(a) XRD spectra of the annealed seed layer and vertically aligned α-MoO_3_ nano-blades on FTO substrates, (b and c) FE-SEM images of vertically aligned α-MoO_3_ nano-blades, (d and e) HRTEM-SAED of α-MoO_3_ nano-blades, (f) Schematic of MoO_3_-nano-blades growth planes on the substrate.

A 100 nm of the MoO_3_ seed layer was directly deposited onto the Cr layer, followed by annealing at 450 °C for 1 h. This leads to the formation of pure α-MoO_3_,^43^ which has been confirmed by X-ray diffraction (Fig. 3a, JCPDS – 005-0508).^12^ A thickness of 100 nm was found to be optimal to uniformly cover the whole substrate, which was directly reflected in the density of MoO_3_ growth (see ESI, Fig. S2†).

### 3.2. Growth of vertically aligned MoO_3_ nano-blades

Vertically aligned MoO_3_ nano-blades were grown directly on the pre-treated seeded substrates under hydrothermal conditions using sodium molybdate as a precursor under strongly acidic conditions. The effect of growth temperature and time on the MoO_3_ structure was systematically analysed by FE-SEM at different growth temperature between 100–200 °C and reaction times between 60–210 min. Prior studies have established that in strongly acidic conditions [MoO_4_]^2−^ ions condensate into hydrated MoO_3_*via* several polymolybdate intermediates.^44^ At elevated pressure and temperature >100 °C the product loses water of crystallisation to form α-MoO_3_ as can be seen in eqn (2):2
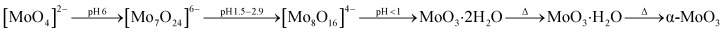


**Fig. 4:**
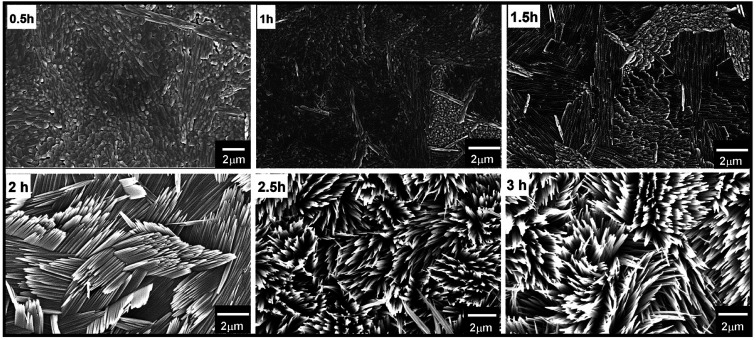
FE-SEM micrographs show the growth of vertically aligned α-MoO_3_ on FTO at different times during the hydrothermal reaction using 0.3 mol L^−1^ acid concentration and reaction temperature of 180 °C.

At optimized precursor concentration and a temperature of 180 °C, the reaction results in a dense array of aligned MoO_3_ nanostructures after 3 h, as shown in Fig. 3b. The XRD analysis of the nano-blades demonstrates that they crystallize in the α-MoO_3_ phase, the same structure as the seed layer. No reflexes of any other phase, impurity or inter-diffusion from the Cr layer could be detected (see Fig. 3a). The sharp reflexes in the diffractogram indicate that the nano-blades are highly crystalline (for the analogous reaction on Si/SiO_2_ substrates see ESI, Fig. S3†). Raman spectra confirmed that the material is pure, stoichiometric α-MoO_3_ (see ESI, Fig. S4†) The XRD pattern was found to have a preferred orientation favouring the (021) planes, which reflects the single crystalline growth along the [001] axis, in a good agreement with previously reported studies.^1,12^ The cross-sectional FE-SEM image (Fig. 3c) confirmed the vertical growth of MoO_3_ nanostructures and arrangement in rows and bundles with a flower-like structure. No by-products from the precursor decomposition appeared as shown in EDX (see ESI Fig. S3†). The individual nano-blades are *ca.* 10–50 nm wide, as shown by HRTEM (Fig. 3d), and consist of fragile 2D sheets that do not stack onto each other. Selected area electron diffraction (SAED, Fig. 3e) recorded perpendicular to the growth axis of the blades is attributed to [010] zone axis which supports preferential growth along the [001] direction (Fig. 3f). The anisotropic crystal growth ties well with the previous study for well-ordered MoO_3_ layers prepared on substrates.^45,46^ It suggests that the nano-blade formation occurs *via* a direct precipitation reaction of highly anisotropic α-MoO_3_.^47^ The nano-blades grow linearly with reaction time until the precursor is wholly consumed or the reaction is interrupted by rapid cooling, as shown in Fig. 4. It is worth mentioning that increasing the hydrothermal reaction time results in overgrowth of the fine structure (see ESI, Fig. S5†). Also, we observe weak adhesion in these overgrown samples, likely due to higher stress triggered by the increased film thickness. This constitutes a significant increase in reaction rate compared to previous studies on hydrothermal synthesis of MoO_3_ nanorods and fibres which give reaction times between 7 h and 7 days respectively.^11,44,48–52^ In our study the growth time is significantly reduced by a factor of 50 of such 2D structures.

We propose that the increasing in the growth rate is triggered by the introduction of our MoO_3_ seed layer, which provides direct nucleation sites for the hierarchical structure growth. Therefore, no-prior time for the formation of nucleation sites is required, allowing a direct initiation of the growth and reducing the growth times considerably (Fig. 4). Another indication for this can be found by carefully looking at the backside of the seed layer (see ESI Fig S6†). Their MoO_3_ crystallites can be observed. Therefore, the nucleation has already occurred before the hydrothermal reaction.

### 3.3. Growth mechanism of aligned MoO_3_ nanostructures

**Fig. 5:**
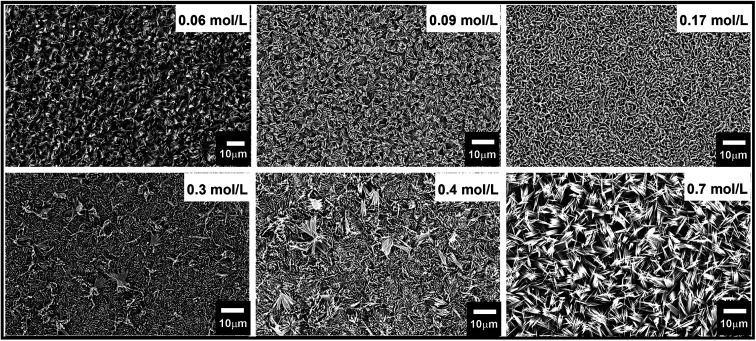
FE-SEM micrographs of vertically aligned α-MoO_3_ nano-blades prepared by varying the acid molar concentration at a growth temperature of 180 °C on the seeded substrate of 10/100 nm of Cr/MoO_3_.

**Fig. 6:**
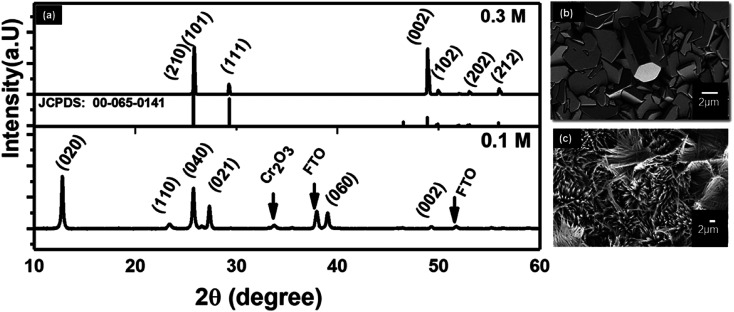
XRD patterns of MoO_3_ nanostructures at a different molar concentration at 180 °C using seed layer of Cr/MoO_3_ of 10/100 nm (a) 0.1 M and 0.3 M. FE-SEM images of MoO_3_ nanostructures at a different molar concentration (b) 0.1 M and (c) 0.3 M.

Hydrothermal synthesis depends on many variables factors which affect the morphology of the resulting nanostructure.^53^ Acid and precursor concentration and reaction time were identified to have the most substantial impact on the immediate reaction. The rate at which the acid was added to the Na_2_MoO_4_ solution to form the reaction precursor was found to be crucial for the shape control of the resulting nanostructures. Slow addition with a rate of ∼1 drop per second yielded thin blades, while a faster rate led to the formation of hexagonal monoliths with a significantly reduced surface area. This highlights the importance of precursor structure and ageing. The absolute amount of acid was used to control the length of the resulting nano-blades with different heights. The isopolymolybdate anions contain octahedral coordinated Mo(vi) ions that are connected *via* edges, as opposed to the tetrahedral coordinated MoO_4_^2^, and thus form a fragment of the α-MoO_3_ target structure. By varying the HCl concentration from 0.06 mol L^−1^ to 0.85 mol L^−1^ the length of the nano-blades could be increased from 3 to 11 μm using a seed layer of Cr/MoO_3_ of 10/100 nm (see Fig. 5, ESI, Fig. S7 and Table S2†). Above a specific concentration of the Na_2_MoO_4_·2H_2_O solution (between 0.02 mol L^−1^ and 0.3 mol L^−1^, depending on the acid concentration) the blade-like shape of the nanostructures changed to larger crystallites with a hexagonal cross-section. Therefore, slow (dropwise) addition of the precursor was necessary to avoid the formation of these larger crystallites. Concomitantly, the crystal structure changed from α-MoO_3_ to h-MoO_3_ (JCPDS 00-065-0141) as can be observed in Fig. 6. The hexagonal phase suffers from a significantly reduced surface area and poor adhesion to the substrates. In this case, we obtain a film thickness of 30 μm with a densely packed structure.

**Fig. 7:**
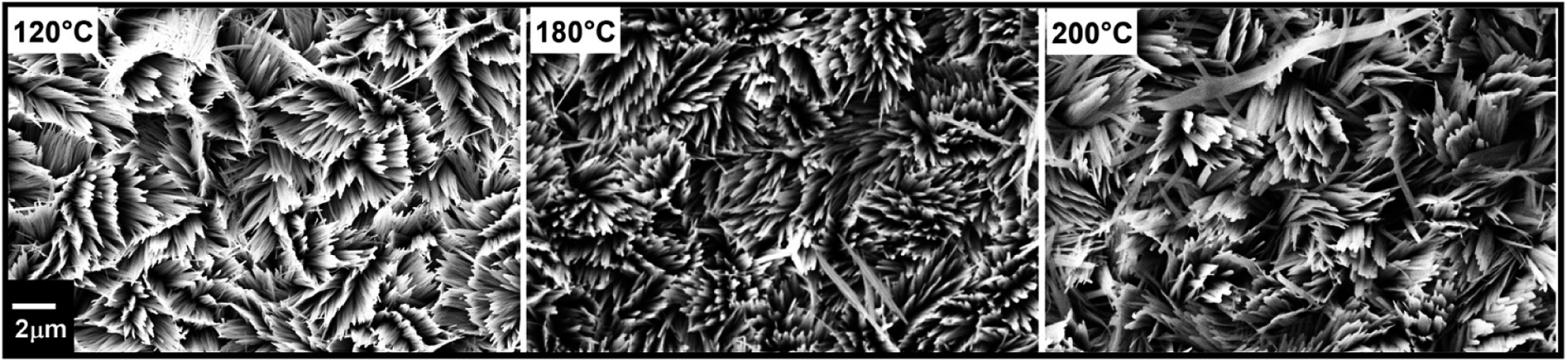
FE-SEM micrographs of vertically aligned α-MoO_3_ nano-blades prepared at different hydrothermal reaction's temperatures using acid molar concentration of 0.3 mol L^−1^ and seed layer of 10/100 nm of Cr/MoO_3_.

The reaction temperature was found to have a minor effect on the morphology between 100 and 200 °C as shown in Fig. 7, in line with previous reports using *in situ* EXAFS, in which the formation of α-MoO_3_ rods above a threshold of 100 °C was observed.^54^

The uniform length of the nanowires and flower-like shapes in which they are arranged on the substrate leads to the conclusion that growth occurs exclusively from the seed particles, and the orientation perpendicular to the substrate is caused by the blades mutually pushing each other up when they start touching (Fig. 4).

While the conditions of the hydrothermal reaction can be tuned to reach optimal conditions for fast growth of a MoO_3_ film with a high surface area, the adhesion layer is crucial to accommodate a wide range of different substrates with significant variations in roughness. Rougher substrates required less Cr deposited for the MoO_3_ film to adhere to the substrate, with FTO requiring a 5–10 nm layer and Si/SiO_2_ requiring 10–20 nm.

## 4 Conclusion

In summary, we have presented a simple, fast, and highly reproducible strategy to grow vertically aligned, crystalline α-MoO_3_ nano-blades on Si/SiO_2_ and FTO substrates under hydrothermal conditions. The synthesis procedure consists of two steps, seed layer formation and seeded growth of highly anisotropic nano-blades by hydrothermal method. The reaction time could be reduced 50-fold compared to literature methods for dispersed α-MoO_3_ nanostructures. The synthesis procedure allows a simple, fast, and large-scale fabrication of vertically aligned nanostructures on different substrates. Reaction time and precursor concentration were found to be the principal parameters to control the shape and aspect ratio of the nano-blades, with a phase transition from orthorhombic α-MoO_3_ to hexagonal h-MoO_3_ occurring at high precursor and acid concentrations. The developed procedure of the vertically aligned nanostructure α-MoO_3_ with high surface area might be favourable for several devices such as gas sensors, supercapacitors, batteries, and others.

## Conflicts of interest

There are no conflicts to declare.

## Supplementary Material

RA-010-D0RA01281E-s001
