# Supplementary material for: Rapid synthesis of vertically aligned α-MoO3 nanostructures on substrates
Source: RSC Adv. 2020 Jun 24;10(40):24119–26. doi: 10.1039/d0ra01281e (PMC9055125; doi:10.1039/d0ra01281e)
Supplement: RA-010-D0RA01281E-s001 [file RA-010-D0RA01281E-s001.pdf]

Electronic Supplementary Material (ESI) for RSC Advances.
This journal is © The Royal Society of Chemistry 2020

Supporting Information

Rapid Synthesis of Vertically Aligned α-MoO_3 Nanostructures on Substrates

Sohaila Z. Noby,[a,b] Ka Kan Wong,[a] R. Ananthakumar,[c] Stephan Siroky,[d] M. Hagner,[a]*

Klaus Boldt,[e] Lukas Schmidt-Mende[a]*

[a]*University of Konstanz, Department of Physics, 78457 Konstanz, Germany*

[b]*National Research centre NRC, Department of solid state of physics,12622 Cairo, Egypt*

[c]SARP-LARPM, *Central Institute of Plastic Engineering and Technology (CIPET), 751024 Bhubaneswar, India*

[d] *University of Konstanz, Department of Chemistry, 78457 Konstanz, Germany*

[e] *University of Konstanz, Department of Chemistry & Zukunftskolleg, 78457 Konstanz, Germany*

Table 1. Lattice parameters of the substrates and the adhesive formed layer of Cr oxides for the calculation of the lattice mismatch.

Material	a (Å)	b (Å)	c (Å)	Ref. Card
FTO (substrate)	4.737	4.737	3.186	00-77-0447
SiO_2 (substrate)	5.082	5.082	7.095	01-086-1561
Cr_2O_3	4.958	4.958	13.593	00-006-0504
Cr_3O	4.544	4.544	4.544	01-072-0528

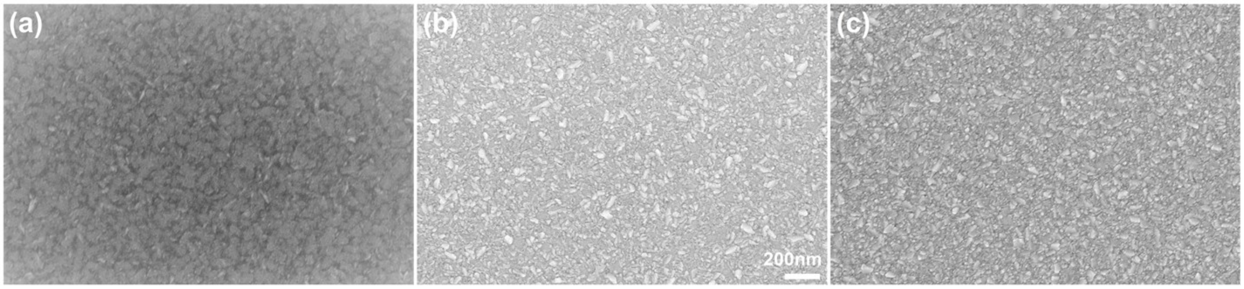

Fig. S1. FE-SEM micrographs of (a) 5 nm (b) 10 nm and (c) 20nm treated Cr evaporated layer.

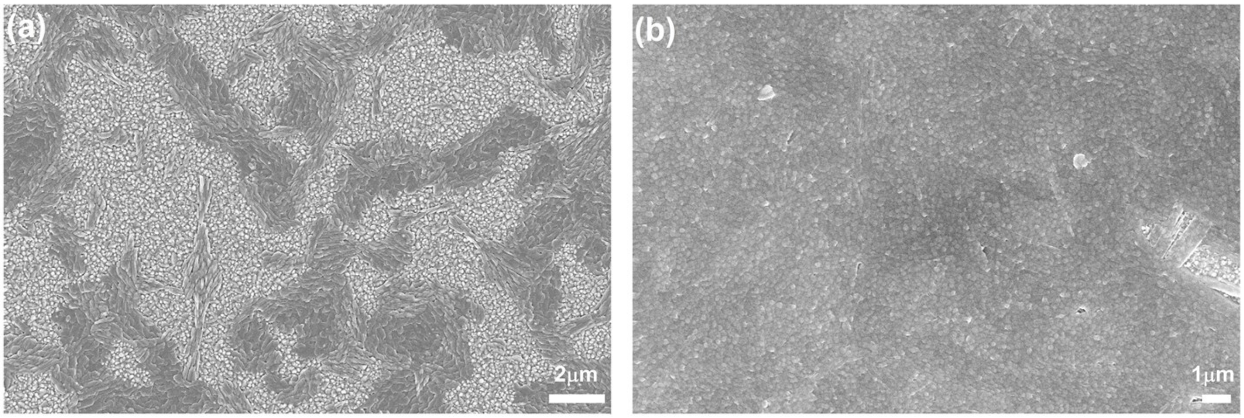

Fig. S2. FE-SEM micrographs of (a) 40 nm and (b) 100 nm evaporated MoO_3 seed layer on FTO substrates.

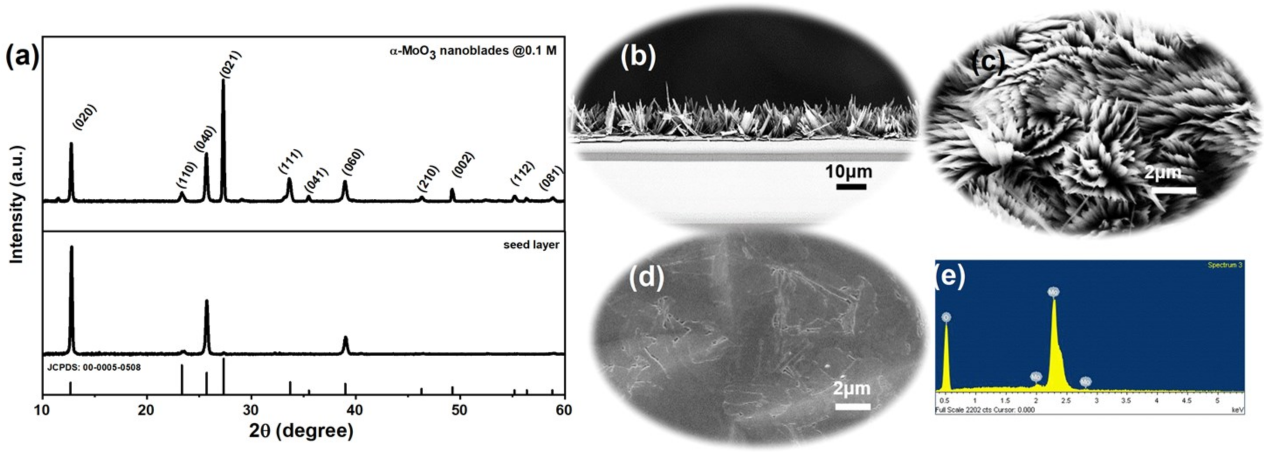

Fig. S3. (a) XRD patterns of seed layer and vertically aligned α-MoO_3 nanoblades on Si/SiO_2 substrate. FE-SEM images of (b) seed layer, (c) cross-section, (d) top view of vertically aligned α-MoO_3 nanoblades on Si/SiO_2 substrate and (e) EDX elemental peaks of the vertically aligned α-MoO_3 nanoblades

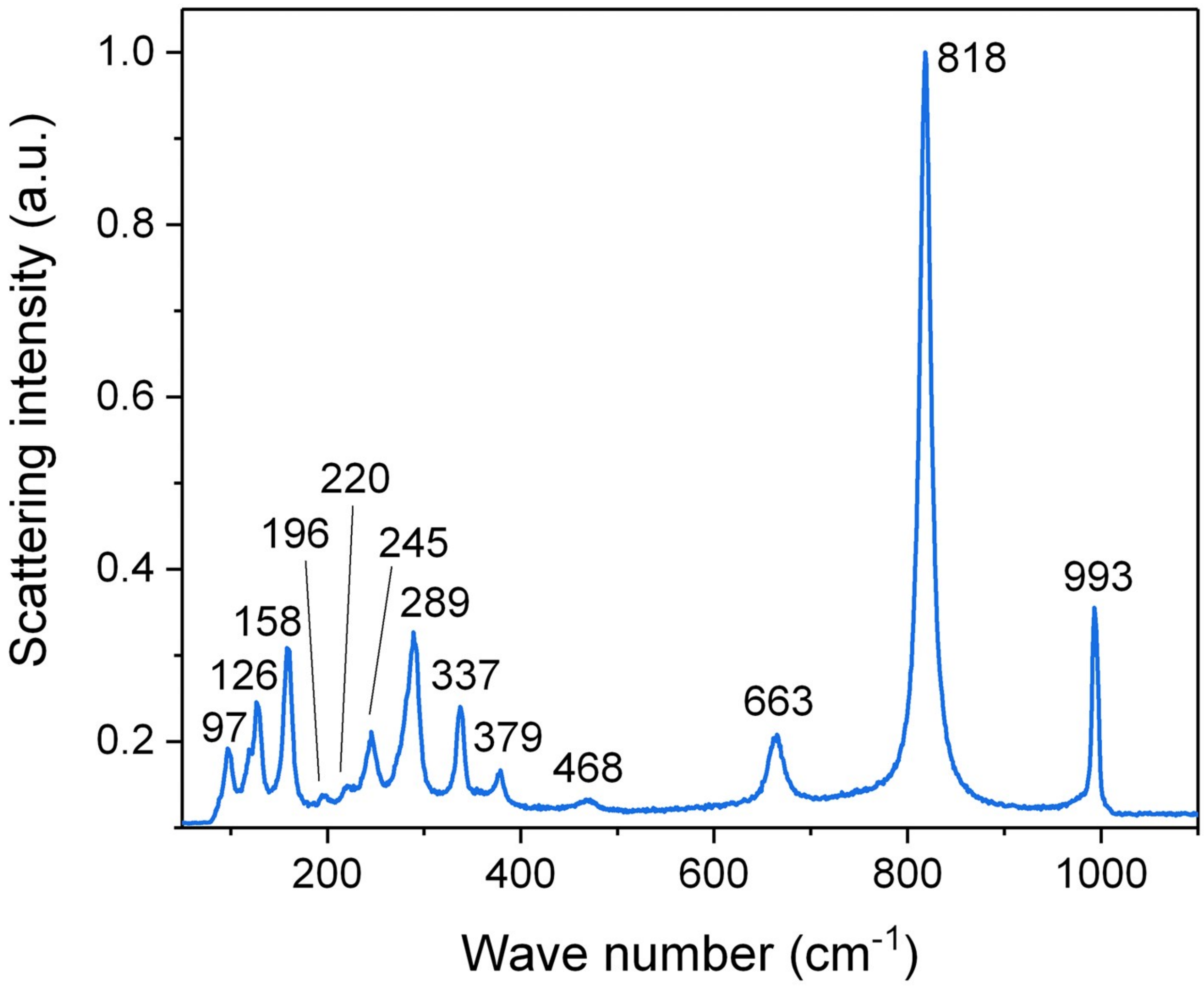

Fig. S4. Raman spectrum of the vertically aligned α-MoO_3 nanoblades recorded using a laser wavelength of 488 nm.

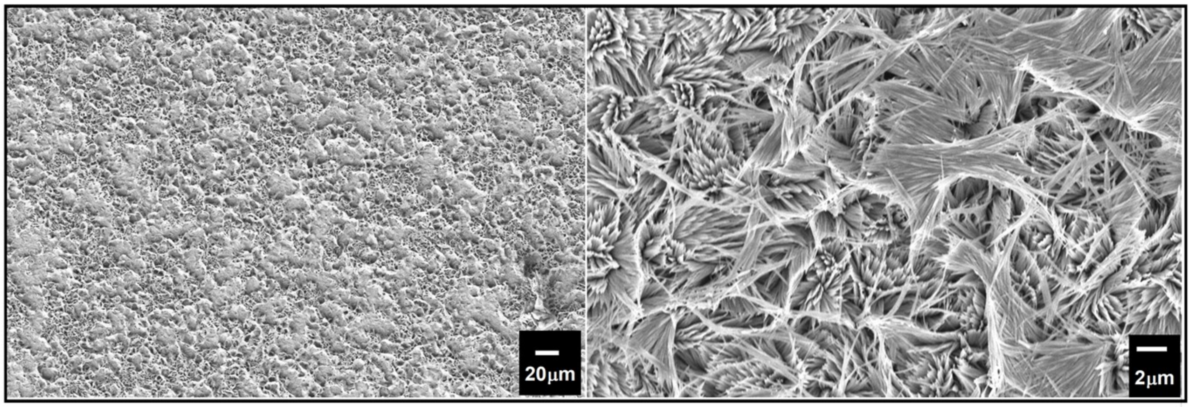

Fig. S5. FE-SEM micrographs show the overgrowth of the vertically aligned α-MoO_3 nanoblades due to an increased hydrothermal reaction time of 4h (using acid concentration of 0.3 mol/L).

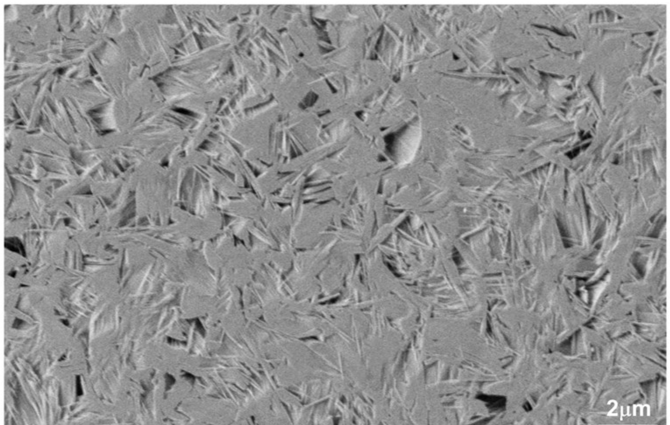

Fig. S6. FE-SEM micrograph of backside of the vertically aligned α-MoO_3 nanoblades after removal from the substrate.

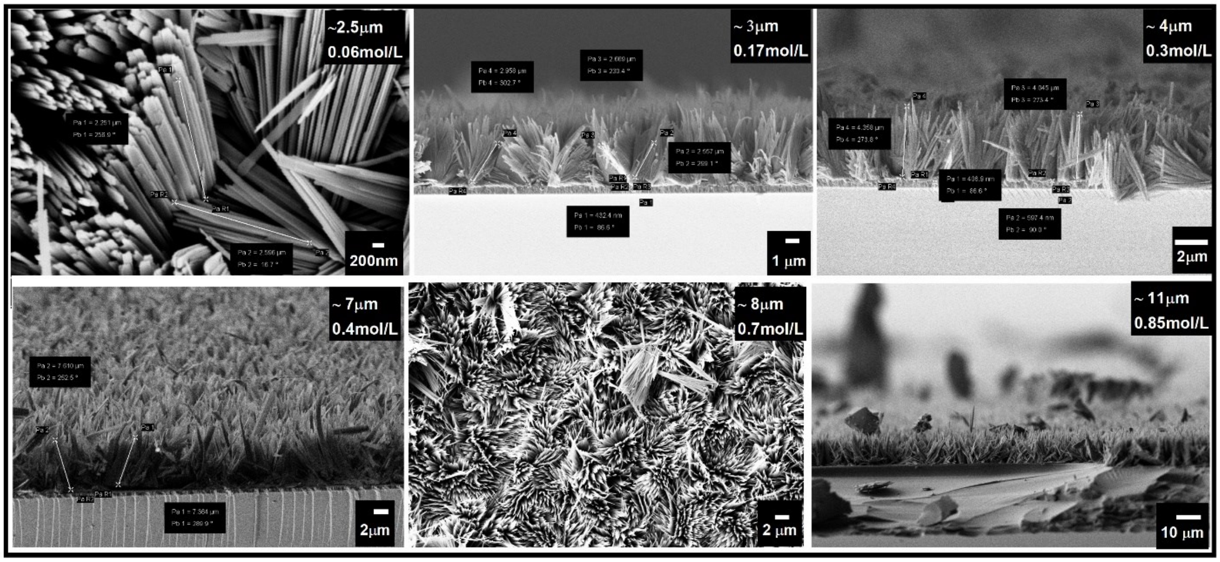

Fig. S7. FE-SEM micrographs show the growth of the vertically aligned α-MoO_3 nanoblades via different acid concentrations on seed layer of 10/100 nm of Cr/MoO_3.

Table 2. The obtained vertically aligned α-MoO_3 nanoblade's length corresponding to the used Acid concentration.

**Acid concentration (mol/L)**	**Vertically aligned α-MoO_3 nanoblade's length(μm)**
0.06	~ 2.5
0.17	~ 3
0.3	~ 4
0.4	~ 7
0.7	~ 8
0.85	~ 11
